# Recent Progress in Physics-Based Modeling of Electromigration in Integrated Circuit Interconnects

**DOI:** 10.3390/mi13060883

**Published:** 2022-05-31

**Authors:** Wen-Sheng Zhao, Rui Zhang, Da-Wei Wang

**Affiliations:** 1 Zhejiang Provincial Key Lab of Large-Scale Integrated Circuit Design, School of Electronics and Information, Hangzhou Dianzi University, Hangzhou 310018, China; davidw.zoeq@hdu.edu.cn; 2 Cadence Design Systems, Inc., San Jose, CA 95134, USA; tangrui.zhang@gmail.com

**Keywords:** integrated circuit interconnects, aging, reliability, electromigration, physics-based modeling

## Abstract

The advance of semiconductor technology not only enables integrated circuits with higher density and better performance but also increases their vulnerability to various aging mechanisms which occur from front-end to back-end. Analysis on the impact of aging mechanisms on circuits’ reliability is crucial for the design of reliable and sustainable electronic systems at advanced technology nodes. As one of the most crucial back-end aging mechanisms, electromigration deserves research efforts. This paper introduces recent studies on physics-based modeling of electromigration aging of on-chip interconnects. At first, the background of electromigration is introduced. The conventional method and physics-based modeling for electromigration are described. Then studies on how electromigration affects powers grids and signal interconnects are discussed in detail. Some of them focus on the comprehensiveness of modeling methodology, while others aim at the strategies for improving computation accuracy and speed and the strategies for accelerating/decelerating aging. Considering the importance of electromigration for circuit reliability, this paper is dedicated to providing a review on physics-based modeling methodologies on electromigration and their applications for integrated circuits interconnects.

## 1. Introduction

Although technology scaling enables integrated circuits (ICs) with higher density and better performance, it is still faced with serious vulnerability to various aging mechanisms appearing from front-end to back-end [1,2,3,4,5,6,7,8,9,10]. These aging mechanisms include Bias Temperature Instability (BTI), Hot Carrier Injection (HCI), Random Telegraph Noise (RTN), Gate-Oxide Breakdown (GOBD) at the front-end, Middle-of-line (MOL) time-dependent dielectric breakdown (TDDB), Back-end-of-line (BEOL) TDDB, and Electromigration (EM). BTI, HCI, RTN, and GOBD cause device parameter deviations. MOL and BEOL TDDBs cause *short circuit* between interconnects, while EM increases interconnects’ resistance and it eventually results into *open circuit*. These phenomena lead to malfunction in circuits. From BTI to BEOL TDDB, there are numerous studies for their impact on device, circuit, and system performance and reliability [11,12,13,14,15,16,17,18,19,20,21,22,23,24,25,26,27,28,29,30], but they are not the topic discussed here. In this paper, our attention is focused on recent progress in physics-based modeling of EM in on-chip interconnects.

**Figure 1:**
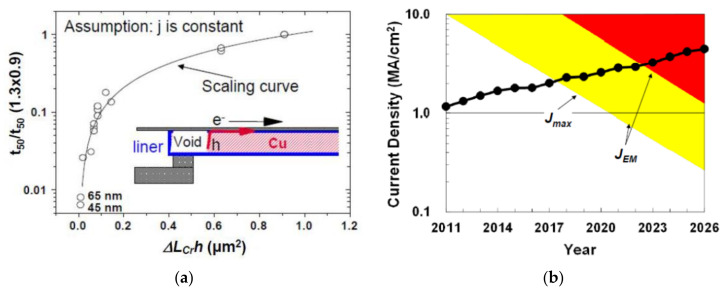
(**a**) Experiment and model of lifetime scaling versus interconnect geometry; (**b**) evolution of *J_max_* (the maximum equivalent dc current density) and *J_EM_* (the current density for target EM life-time). They are predicted by ITRS-2015 [31].

EM is the migration of interconnects’ metal atoms after they obtain momentum from moving electrons. Since the interconnects are wrapped by barrier layer, the metal atoms’ movement causes depletion regions and it, eventually, causes void nucleation and growth. EM induced degradation and failure is one of the most critical reliability issues for deeply scaled ICs. Such a degradation is expected to get even worse with further technology scaling. It is reported by ITRS-2015 that the operating current density has exceeded 1 MA/cm^2^ and is rapidly approaching to 10 MA/cm^2^. Figure 1a shows the experiment and model of lifetime scaling versus interconnect geometry, where an effective scaling model has been established by assuming the void is located at the cathode end of the interconnect which contains a single via with drift velocity dominated by interfacial diffusion [31]. The interconnects’ EM lifetime is predicted to decrease by half for each new generation. Figure 1b shows evolution of *J_max_* (the maximum equivalent dc current density) and *J_EM_* (the current density for target EM lifetime) [31]. Both *J_max_* and *J_EM_* are limited by interconnect geometry. *J_max_* increases with the increase of operating frequency and the reduction of interconnect cross-section. Although there are several process options, such as usage of Cu alloys seed layer and the short length effect to overcome EM severity, EM is still an inescapable topic since the operating current density is entering *J_EM_* region, as shown in Figure 1b. It is necessary to apply novel models and methodologies to study the strategies which can help mitigate EM degradation by incorporating the innovations in material and process.

Depending on their functionality, on-chip interconnects can propagate signal within and between cells and deliver power to sub-circuits. Most of the previous EM studies have focused on the reliability of power grids and clock/signal nets. In traditional approach, the Blech limit and Black’s equation are applied to investigate interconnects’ EM reliability. The Blech limit or Blech product is [32]

(1) $$\left(j\times L\right)\leq {\left(j\times L\right)}_{crit}=\frac{\Omega \sigma_{crit}}{eZ\rho }$$

where *j* is the current density, *L* is the interconnect branch length, Ω is the atomic volume, *σ_crit_* is the critical stress for void nucleation, *e* is the electron charge, *eZ* is the effective charge of migrating atoms, and *ρ* is the metal resistivity. 

The interconnect branches with (*j × L*) ≤ (*j × L*)*_crit_* are filtered out as EM immortal. The mean time to failure of remaining EM mortal branches is characterized with Black’s equation [33]

(2) $$MTTF=Aj^{-n}exp\left\{E_{a}/k_{B}T\right\}$$

where *A* and *n* are assumed to constants, *E_a_* is the activation energy, *k_B_* is the Boltzmann’s constant, and *T* is the absolute temperature. 

*A* and *n* are measured through accelerated test with a higher current density injected under higher temperature. Then the *MTTF* under normal use condition is extrapolated as a function of *MTTF* under accelerated test,

(3) $$MTTF_{use}=MTTF_{stress}{\left(\frac{j_{stress}}{j_{use}}\right)}^{n}exp\left\{\frac{E_{a}}{k_{B}}\left(\frac{1}{T_{use}}-\frac{1}{T_{stress}}\right)\right\}$$

where *j_stress_* and *T_stress_* are current density and absolute temperature under accelerated test, while *j_use_* and *T_use_* are current density and absolute temperature under normal-use condition, respectively. 

**Figure 2:**
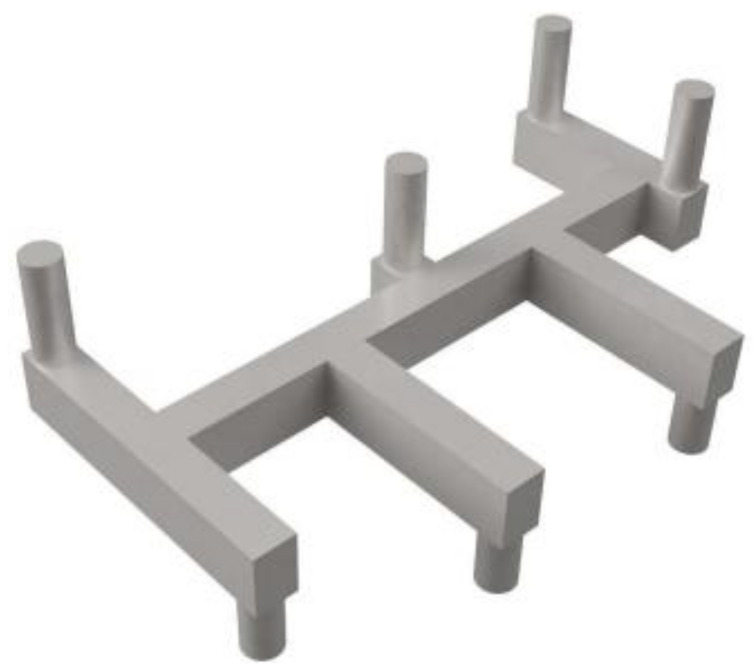
An interconnect tree with multiple segments confined by diffusion barriers/liners in one-layer metallization [34].

Based on *MTTF_use_*, the probability of failure (at a specific stress time) of each branch is obtained from cumulative distribution function of its lifetime distribution (lognormal or weibull). Then the overall probability of failure of studied branches is computed by weakest-link statistics. Although this traditional approach is convenient to use, it does not provide accurate estimation on EM failure due to the following reasons: first, n is not really a constant under different current density especially when *j_stress_* is much larger than *j_use_*; second, the Belch limit is accurate only for single branch interconnect. It is inapplicable for a general interconnect tree with multiple segments/branches. As shown in Figure 2, since the segments are connected without barriers, it is necessary to consider the atom transportation between them [34]. Third, weakest-link statistics considers a single branch failure leads to failure of studied interconnect trees which is not true in state-of-the-art ICs, especially for power grids with a mesh structure [35]. Therefore, some physics-based EM compact models have been proposed to incorporate the atom movement between segments [36,37,38,39,40,41,42,43,44]. It is noted that there are already several excellent reviews on relevant topics in [34,45,46,47,48], but they are mainly on power grids. In this review article, we will summarize the studies on physics-based EM compact modeling and its applications on various on-chip interconnects and their most recent updates. The remainder of this paper is organized as follows. Section II introduces the physics-based three-phase compact EM model. The model should be imposed on interconnects with corresponding current density and boundary conditions. The applications of this compact EM model in current and future interconnects are discussed as well. Section III summaries recent progress in EM studies mainly on power grids. These studies cover from Black’s equation-based simulations to physics-based simulations. Section IV shows the application of physics-based modeling on interconnects in cache memory. The impact of physical dimensions and cache configurations on EM reliability is discussed. Section V concludes this paper.

## 2. Physics-Based Three-Phase Compact Model

**Figure 3:**
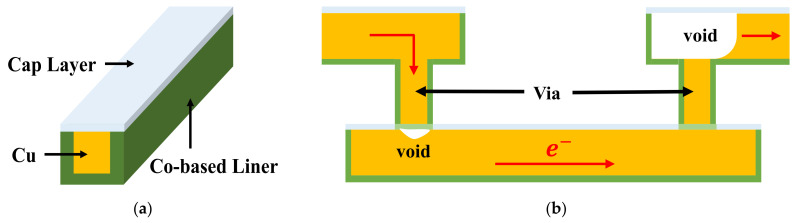
(**a**) Cu dual damascene conductor structure, where the trench is lined with a Cobalt-based liner and the Cu is capped with either a dielectric or metallic layer. (**b**) Void formation in a Cu wire connected with a via-below (left) and a via-above (right) [49].

EM is the phenomenon of metal atoms migration due to the momentum obtained from electrons when there is current flowing over interconnects. Since the momentum is also transferred between metal atoms, hydrostatic stress would appear in the interconnects confined by barrier material and capping layer. Although the stress gradient may prevent atoms to move, the maximum stress continues to increase until it reaches the saturation value or a critical stress value for void generation. A void is considered to be nucleated at the position where the maximum hydrostatic stress reaches critical stress value. Then the void is incubated and it grows under further stimulus by electrons’ movement. A void will never appear in an interconnect if the saturation stress value is smaller than the critical value. Such an interconnect is EM immortal. Figure 3a shows a Cu dual damascene conductor structure [49]. The trench is lined with a Cobalt-based liner and the Cu is capped with either a dielectric or metallic layer. Figure 3b shows the voids possibly formed in an interconnect. The void formed under a via can cause *early failure* when the size is large enough to cover the via bottom and it leads to an open circuit status, while the void formed above a via causes *conventional failure* when the gradually growing void introduces an obvious resistance shift which causes circuit’s functional failure with a significant performance deviation. Both *early* and *conventional failures* need to be checked during EM analysis. The EM process during void generation and growth can be described with a three-phase model which consists of nucleation phase, incubation phase, and growth phase. To calculate the time-dependent EM process, Korhonen’s model has been proposed to capture the time-varying stress. The Korhonen’s model together with appropriate initial condition and boundary conditions are able to accurately emulate hydrostatic stress evolution in interconnects. Thereafter, the time-dependent stress and atomic flux are combined to obtain resistance shift due to EM. The resistance shift can be inserted into circuit simulation to show its impact on performance. This section introduces Korhonen’s model and the three-phase compact EM model. 

### 2.1. Korhonen’s Model

In general, multi-physics 3-D simulation is able to present accurate estimation on hydrostatic stress and resistance shift in interconnects. However, since it requires vast computation resource and the simulation speed gets very slow when the studied interconnect structure (such as power grids for a microprocessor) is relatively large, 3-D numerical simulation is not always preferred for EM analysis. Fortunately, Korhonen’s 1-D model offers a reasonable trade-off between accuracy and efficiency. Let us take a branch with a length of *L* as an example. The evolution of 1-D hydrostatic stress follows [50]

(4) $$\frac{\partial \sigma }{\partial t}=\frac{\partial }{\partial x}\left[\frac{D_{a}B}{k_{B}T}\left(\Omega \frac{\partial \sigma }{\partial x}-eZ\rho_{Cu}j\right)\right]$$

(5) $$J_{a}=\frac{D_{a}C_{a}\Omega }{k_{B}T}\left[\frac{\partial \sigma }{\partial x}-\frac{eZ\rho_{Cu}j}{\Omega }\right]$$

where the effective atomic diffusivity $D_{a}=D_{0}exp\left(-E_{a}/k_{B}T\right)$. *D_0_* is a constant, *E_a_* is the effective activation energy, $k_{B}$ is Boltzmann’s constant, *T* is absolute temperature, *B* is the effective bulk elasticity modulus, Ω is the atom lattice volume, eZ is the effective charge of migrating atoms, $\rho_{Cu}$ is copper resistivity, *j* is the current density over the studied interconnect, $C_{a}$ is the atom concentration, $J_{a}$ is the atomic flux, and *t* is the stress time.

Initial conditions and boundary conditions are necessary for a solution of hydrostatic stress. They are decided by the material difference in the coefficients of thermal expansion (*CTE*) and the difference between stress free annealing temperature and the circuit temperature. The position dependent (*x* ranges from *0* to *L*, *x* = *0* is at the branch’s left end, and *x* = *L* is the branch’s right end when it is assumed to be placed horizontally) initial value (at *t* = 0) is expressed as [51]

(6) $$\sigma \left(x,t=0\right)=B\left(a_{M}-a_{Conf}\right)\left(T_{ZS}-T\left(x,t=0\right)\right)$$

where *T_ZS_* is the stress free annealing temperature, *x* denotes the node position, *T*(*x*, *t* = 0) is the specific node temperature at *t* = 0, and *a_M_* and *a_Conf_* are the *CTE* of the metal and confinement materials, respectively.

The studied branch may be connected to other branches at its left and right ends. The boundary conditions at the two ends depend on whether they are connected to other branches and whether the voids have appeared at their locations. At the left and right ends, the boundary conditions before and after void nucleation are given as [41,44]

(7) $$\left\{ \begin{array}{c} \overset{K_{l}}{\underset{k=0}{\sum }}J_{a,\;k}w_{k}h_{k}=0,\;for\;left\;node\;if\;it^{\prime }\;in\;nucleation\;phase\\ \frac{\partial \sigma \left(x=0,t\right)}{\partial x}=\frac{\sigma \left(x=0,t\right)}{\delta },\;for\;left\;node\;if\;it^{\prime }s\;in\;incubation\;and\;growth\;phases\\ \overset{K_{r}}{\underset{k=0}{\sum }}J_{a,\;k}w_{k}h_{k}=0,\;for\;right\;node\;if\;it^{\prime }s\;in\;nucleation\;phase\\ \frac{\partial \sigma \left(x=L,t\right)}{\partial x}=-\frac{\sigma \left(x=L,t\right)}{\delta },\;for\;right\;node\;if\;it’s\;in\;incubation\;and\;growth\;phases \end{array}\right.$$

where *K_l_* and *K_r_* are the total number of branches connected to the left and right nodes, respectively, *w_k_* and *h_k_* are the width and thickness of their *k_th_* connected branch, and *δ* is the thickness of the void surface. It is noted that the maximum hydrostatic stress (during void nucleation) in one branch appears at its boundary nodes and the void may not appear at the boundary nodes if the hydrostatic stress at their position never exceeds critical stress (*σ_th_*), then the boundary condition should be always applied as the one in nucleation phase.

### 2.2. Three-Phase Compact Model

**Figure 4:**
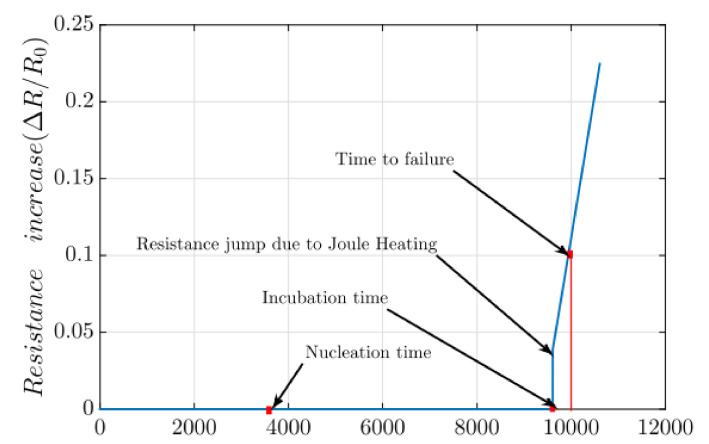
Resistance change over time under the three-phase EM model [45].

The three-phase compact model includes nucleation, incubation, and growth phases, as shown in Figure 4 [45]. When the interconnect branches are flown over by currents, the hydrostatic stress on them evolves with the initial value described in Section 2.1. In the nucleation phase, the resistance of a studied branch remains unchanged before the maximum stress exceeds a threshold (*σ_th_*), while after it exceeds the threshold, the model enters the incubation phase where the void size is increasing but it is still smaller than a threshold size. The interconnect resistance is stays unchanged in nucleation and incubation phases. Later, the model enters growth phase if the time-dependent void size grows large enough to cover the interconnects’ cross section. The resistance jump is caused by Joule heating. In the growth phase, the branch’s time-dependent resistance shift can be expressed as [49]

(8) $$\Delta R=L_{v}\left(t\right)\left(\frac{\rho_{liner}}{\left(W+2H+2t_{liner}\right)t_{liner}}-\frac{\rho_{cu}}{WH}\right)$$

where *W* and *H* are the width and height of the studied branch, respectively, *ρ_liner_* and *t_liner_* are the resistivity and thickness of the liner. *L_v_*(*t*) is the time-dependent void length, and its growth can be calculated with Δ*L_v_*(*t*) *= J_a_*(*t*)·Ω·Δ*t*.

**Figure 5:**
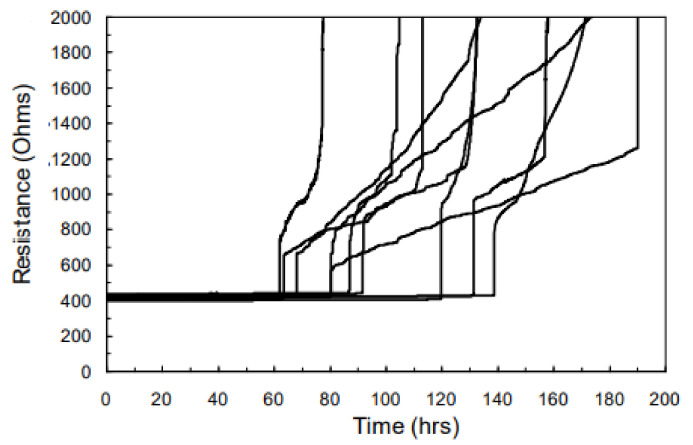
Resistance trace of interconnects under EM testing [52].

The three-phase model can be validated by experimental data. Figure 5 shows resistance trace of interconnects in experimental measurements [52]. Obviously, most of the traces follow resistance shift behavior described by the three-phase EM model. 

### 2.3. Model Applications

The Korhonen’s model describes time-dependent evolution of hydrostatic stress in specific branch. Since the interconnects are confined by diffusion barriers/liners in one-layer metallization, the whole interconnect network can be divided into many individual interconnect trees which are similar as the one shown in Figure 2. For interconnect tree, their hydrostatic stress evolution can be evaluated independently. It should be noted that the current density over the branches may vary under the appearance of resistance shift of some branches. For each branch in a specific tree, the Korhonen’s model can be applied with appropriate initial condition and boundary conditions to accurately compute the hydrostatic stress evolution. Based on this, the time-dependent resistance shift of the EM mortal branch is obtained conveniently. The model has been applied to check the reliability of state-of-the-art Cu interconnects. More details are given in the following sections. With the continuous scaling, the Cu interconnects in sub-10 nm technology node suffer from high resistance due to serious surface scattering of the electrons flowing over them. It is found in [53,54] that reducing the linewidth to 10 nm results in a drop of *j_max_* to below 1 MA/cm^2^ and scaling linewidth from 25 nm to 10.5 nm leads to a 90% drop of *j_fail_* i.e. the current density that induces failure at 10 y. Under such a scenario, interconnects based on Ru and Co are potential replacements with better reliability than Cu because of their lower resistivity and higher EM activation energies [55,56,57,58,59,60]. The barrierless Ru interconnect together with an integration scheme have been identified to be more EM reliable than Cu [61]. And it is found that full Ru vias have no risk of voiding after long thermal storage at high temperature [62]. With respect to Co interconnects, the first estimation of an effective D_0_Z* (~1.72 × 10^−10^) for Co is performed [63]. It is two orders of magnitude lower than Cu. While Co vias may be EM immortal, the lack of a barrier may induce diffusion along the Co/dielectric interfaces and Co/Cu intermixing [64]. The physics-based compact EM model introduced in this section is also applicable to interconnects based on new materials such as Co and Ru only if the diffusion coefficients and microstructures are extracted from experiment result [65]. 

## 3. Modeling of EM Impact on Power Grids

In the most previous studies of EM modeling, the Black’s equation and Blech limit are applied to analyze the reliability of signal interconnects and power grids [66,67,68,69,70,71,72,73]. In [66,67,68], by incorporating the Joule heating effect, Gracieli Posser et al. have developed approaches for modeling and efficient characterization of cell-internal EM and have simulated EM effects on different metal layers at different wire lengths. On the one hand, they found the cell-internal EM reliability can be optimized with layout modification and constraints on output pin position. On the other hand, it is concluded that larger metal layers have smaller EM effects and, consequently, a higher EM lifetime for the wires. Palkesh Jain et al. proposed a SoC-level logic-IP-internal EM verification methodology which provides on-the-fly retargeting capability for reliability constraints [69,70]. The proposed approach is demonstrated on a 28-nm design. Meanwhile, they presented a fast and stochastic analysis methodology to overcome the lifetime under-estimation by conventional methodologies based on weakest-link assumption for EM assessment of click grids and power grids [71,72]. Vidya et al. also applied Black’s equation and Blech limit to estimate the self-heating impact on EM reliability of FinFET and GAAFET designs [73]. In order to overcome the reliability under-estimation due to the traditional series model for EM checking and the pessimistic assumptions about the chip workload and the corresponding supply currents, Mohammad Fawaz and Sandeep Chatterjee et al. proposed a framework for EM checking that allows users to specify conditions-of-use type constraints which help capture realistic chip workload and which includes the use of a novel mesh model for EM prediction in the grid, instead of the traditional series model [74,75,76]. They developed a framework to estimate the change in statistics of interconnects as their effective-EM current varies and developed a novel vector less mesh model technique to estimate the average minimum time-to-failure of a power grid under uncertain workload. Their results indicate that the series model causes pessimistic estimation of power grid MTF and reliability by a factor of 3–4 [75,76]. 

Although the novel methodologies/frameworks based on Black’s equation can estimate EM reliability, since the atom flow within segment trees is ignored there, they are not able to provide suitable and accurate results as physics-based methods. Sandeep Chatterjee et al. found that the power grid’s lifetime estimated by their physics-based approach is on average 2.75× longer than those based on Black’s model [44]. Xin Huang et al. verified that the lifetime of IBMPG2 predicted by the traditional approach with the series and mesh model is 7.82 y and 10.67 y while the lifetime predicted by a physics-based EM model is 15.66 y [37]. It means the frameworks based on conventional estimation method is too conservative, therefore, the physics-based EM model is preferred for designs with space limited. In this section, our attention is focused on the studies which applied physics-based model to investigate the EM impact on power grids. This section consists of three subsections. The first subsection covers modeling and simulation methodologies proposed to study power grids’ reliability. The second subsection focuses on the strategies adopted to improve EM evaluation speed while ensuring good accuracy and the optimization methodologies for better EM reliability. The third subsection is mainly on techniques for EM acceleration and deceleration.

### 3.1. Modeling and Simulation Methodologies

**Figure 6:**
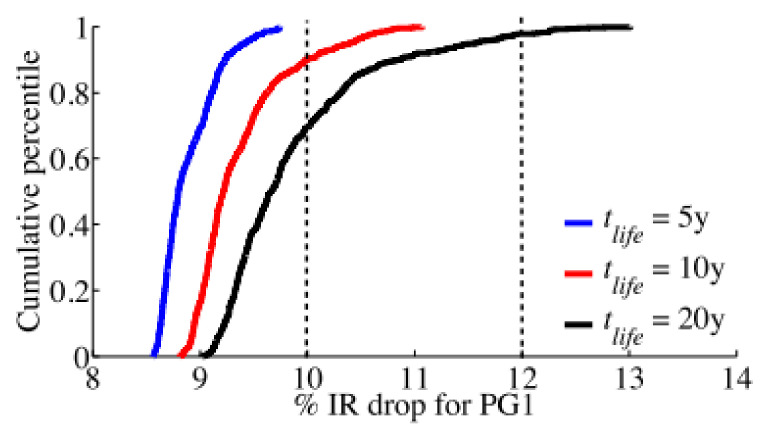
CDF plots for IR drop of a studied power grid for different circuit lifetimes, t_life_ [40].

**Figure 7:**
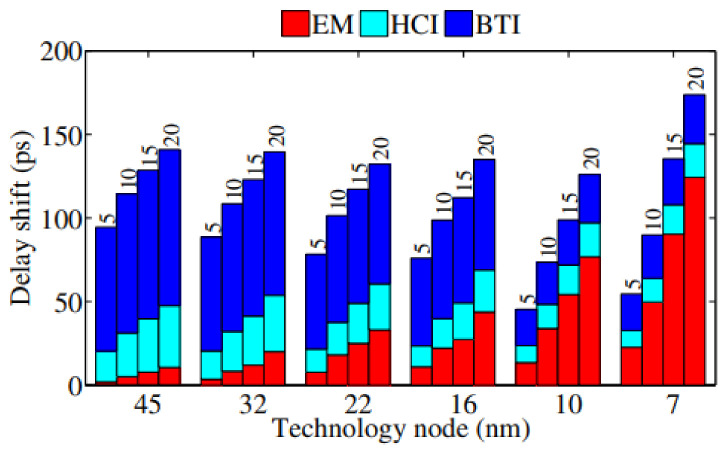
Absolute delay shifts due to various aging mechanisms for advanced technology nodes at circuit operation time of 5, 10, 15, and 20 y [38].

There are many studies on modeling and simulation methodologies for EM reliability of power grids with applying physics-based models. Some of them are based on numerical simulations while the others are based on analytical solutions. At an early time, Vivek Mishra et al. modeled the impact of EM in Cu interconnects on power grid integrity with using probability analysis [36,38,39,40,43]. Figure 6 shows the CDF plots for IR drop of a studied power grid for different circuit lifetime [40]. For t_life_ = 5 y, the studied power grid remains functional because all samples’ IR drop are under a 10% threshold, however, it has a worst resistance degradation of 48% which is much more than a typical 10%~20% resistance increase criteria used by circuit designers. It verifies that power grids have inherent resilience to EM failures. They also studied circuit delay variability due to interconnects’ resistance shift under AC EM [38]. It shows that even non-catastrophic EM on critical paths can cause serious performance degradation which ultimately result into circuit malfunction. As shown in Figure 7, the impact of EM on absolute delay shifts increases under technology scaling [38]. It is mainly because the higher current density in smaller interconnects exacerbate EM degradation. Under a specific technology node, EM effect becomes more obvious because EM void size and number increase with stress time. Meanwhile, they developed methods to evaluate transient stress evolution in interconnects, and presented simple and practical criteria for EM mortality checking. It is demonstrated that the number of EM mortal interconnects highly depend on lifetime target and reliability expectations [39]. It is also observed in [43] that power grids’ EM degradation is impacted by configuration of via arrays which connect interconnects at different layers.

**Figure 8:**
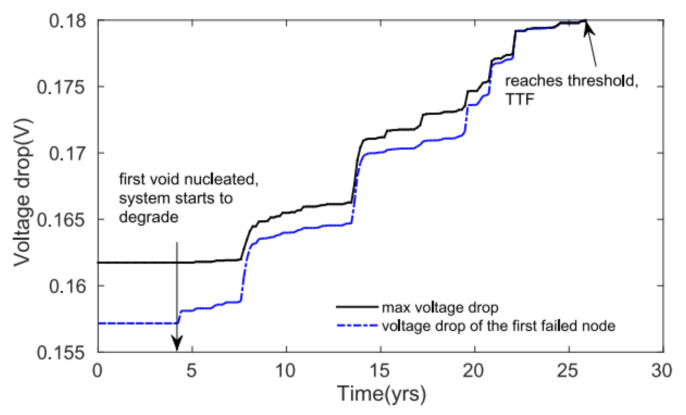
Time-dependent voltage drop of the first failed node and maximum voltage drop in IBMPGNEW1 [42].

In order to accurately model EM degradation in power grids, Xin Huang et al. proposed a new physics-based assessment method [37,42]. This method incorporates power grids’ redundancy by assuming the circuits get failed only when the IR drop reaches a threshold value. The hydrostatic stress in each interconnect tree is evaluated with considering atom transportation between connected branches. The experimental result not only verifies that the result obtained from Black’s equation is too pessimistic, it but also shows that IBM P/Gs’ EM failure is more likely to happen at the places with large initial stress value and is more likely to happen at longer time when the void volume saturation phenomenon is taken account. The time-dependent IR drop can be captured by placing the EM induced resistance shift into the P/G circuit model. Figure 8 shows the time-dependent voltage drop of the first failed node and maximum voltage drop in IBMPGNEW1 [42]. The proposed method is also applied to study the impact of cross-layout temperature and thermal stress distributions on full-chip EM assessment. 

Since most of the previous studies are based on the uniform temperature assumption, Xin Huang et al. implemented a flow of EM assessment for multi-layer P/G in a 32 nm test chip [77,78]. The cross-layout temperature variation due to devices’ power consumption and interconnects’ Joule heating are characterized and incorporated into EM assessment. It is found that uniform temperature assumption causes inaccurate prediction on TTF and the thermal stress variation results into better evaluation on EM reliability. It means the on-chip temperature variation is needed to get more reasonable EM assessment results. With applying the same physics-based EM model, Kai He et al. proposed a lightweight on-chip aging sensor [79]. The interconnects in this sensor are designed to have detectable EM failure at specific time. A number of parallel interconnects are used in the sensor to mitigate inherent variations. The EM-based aging sensor can provide more accurate prediction of chip usage time and offers simpler circuit implementation and smaller area footprints than the ring-oscillator based sensor. Chase Cook et al. has applied finite difference method to solve 1-D EM problem in multi-branch interconnects [80]. The new method can easily accommodate non-uniformly distributed residual stress and time-dependent temperature and current during circuit operation. The numerical results match well with that of COMSOL which is based on finite element method. Taeyoung Kim et al. presented an approach for system-level EM reliability management for multi/many core microprocessors [81]. They proposed a task migration method to balance EM resource consumption by all the cores. It treats TTF as a resource to consume during task execution and uses task migration to balance TTF consumption across the cores. It equalizes the probability of failure of each core to maximize the lifetime of multi-core system. The simulation results show a balanced TTF consumption by the cores and the system’s EM reliability has been maximized. 

**Figure 9:**
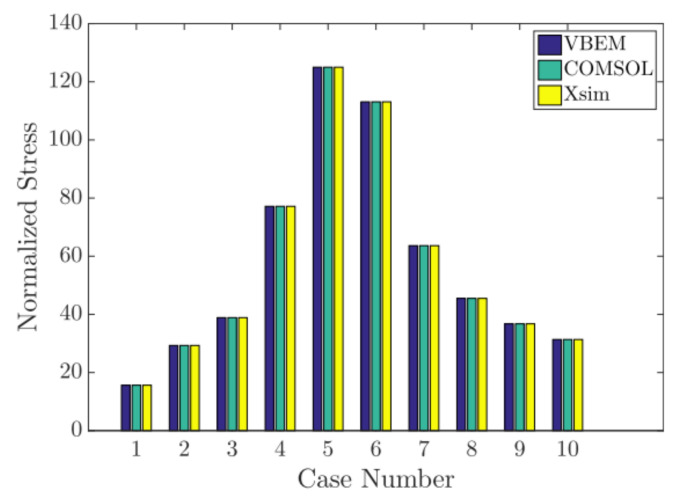
Steady-state EM stress comparisons for each straight-line 3-terminal interconnect case [82].

**Figure 10:**
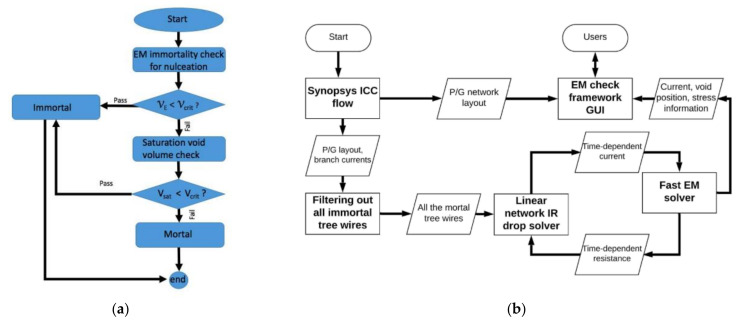
(**a**) EM immortality check algorithm flow, and (**b**) simulation framework for *EMSpice* simulator [83,84].

Since the current in P/G is unidirectional, the interconnects’ EM immortality can be determined by checking steady-state stress distribution. If the maximum stress is larger than critical value, the void is nucleated in a mortal interconnect, otherwise, the interconnect is immortal. However, since there is not closed form for steady state stress in multi-branch interconnect trees, numerical solutions generally need long time simulation. New techniques for convenient immortality checking are necessary for EM study. In [82], Zeyu Sun et al. proposed a new parameter called Critical EM voltage (*V*_Crit,EM_) to evaluate EM immortality at steady-state stress in multi-branch interconnect tree. The *V*_Crit,EM_ is an extension of Belch limit concept. The difference is that Blech limit is for single branch while *V*_Crit,EM_ is applicable to multi-branch tree. Since this voltage-based EM (VBEM) method overcomes the problem of current-density-based criteria, it can handle the impact of interconnect tree structure on EM-induced stress with easy implementation. With this method, the EM voltage at the ground node or cathode node of a tree is determined with the total area of branches in the tree, the total area of the branches connected to each node in the tree, and the nodal voltage at each node. The EM immortality of studied tree depends on whether the EM voltage is smaller than the *V*_Crit,EM_. This criterion is applicable for immortality checking in void nucleation phase. The VBEM analysis not only agrees with results from finite difference method at steady state but also matches well with COMSOL and XSim results, as shown in Figure 9. Since the VBEM analysis assumes that current density is evenly distributed in one branch, the impact on current crowding is investigated. Comparison with COMSOL result shows that current crowding effect is unobvious if the length of a branch is much greater than its width. Void saturation volume is another important issue for EM immortality checking. Since previous saturation volume model only works for single branch, Zeyu Sun et al. derived a void saturation volume model for multi-branch tree, as shown in Figure 10a [83]. The model follows atom conservation at steady state of void growth phases. The overall void saturation volume in a tree at steady state is the sum of saturation volume contributed by each branch. The tree is considered as EM immortal if the overall void volume is smaller than the critical size. This criterion is applicable for immortality checking in void incubation phase. Transient analysis is necessary on the tree only when it is EM mortal. The authors proposed a new EM immortality checking flow which considers the checking criteria in both void nucleation phase and void incubation phase. The algorithm is given in Figure 10b. The new flow reduced conservativeness of existing EM assessment methods, and it helps the designer quickly identify the new type of immortal branches which have void nucleated but with a size smaller than the critical value.

Based on their EM immortality check algorithm shown in Figure 10a and the existing transient EM analysis theories, Zeyu Sun et al. developed a new full-chip EM simulator called *EMSpice* to evaluate EM reliability of P/Gs [84]. Figure 10b shows the simulation flow of *EMSpice*. It starts from P/G layout information from Synopsys IC Compiler. In the first step, it disregards immortal trees by considering the immortality criteria in nucleation and nucleation phases. Then, the mortal trees are applied with a FDTD solver to extract time-dependent hydrostatic stress in both nucleation and post-voiding phases. Since the EM-induced resistance shift cause current variation in P/G, the EM analysis is interacting with IR drop analysis of a whole P/G at each time step to ensure the comprehensiveness of *EMSpice*. *EMSpice* simulator can reduce the over-conservation in EM assessment. It predicts the failed tree number 76.7% less than the Black’s method and 66.7% less than another full-chip EM analysis method. 

**Figure 11:**
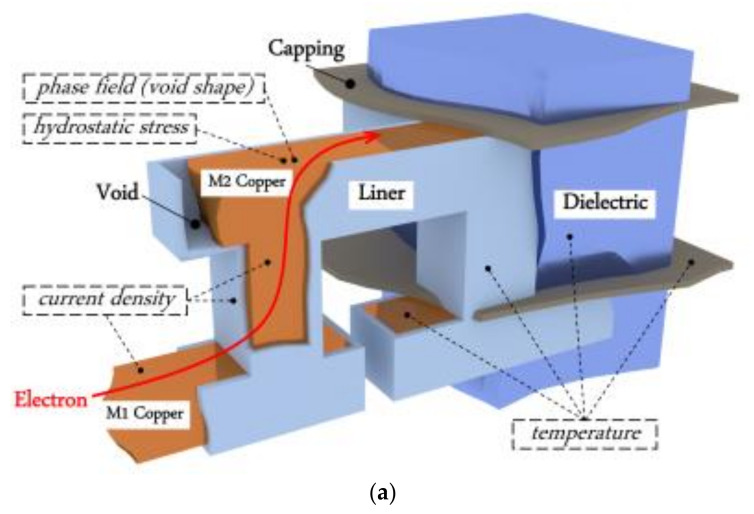

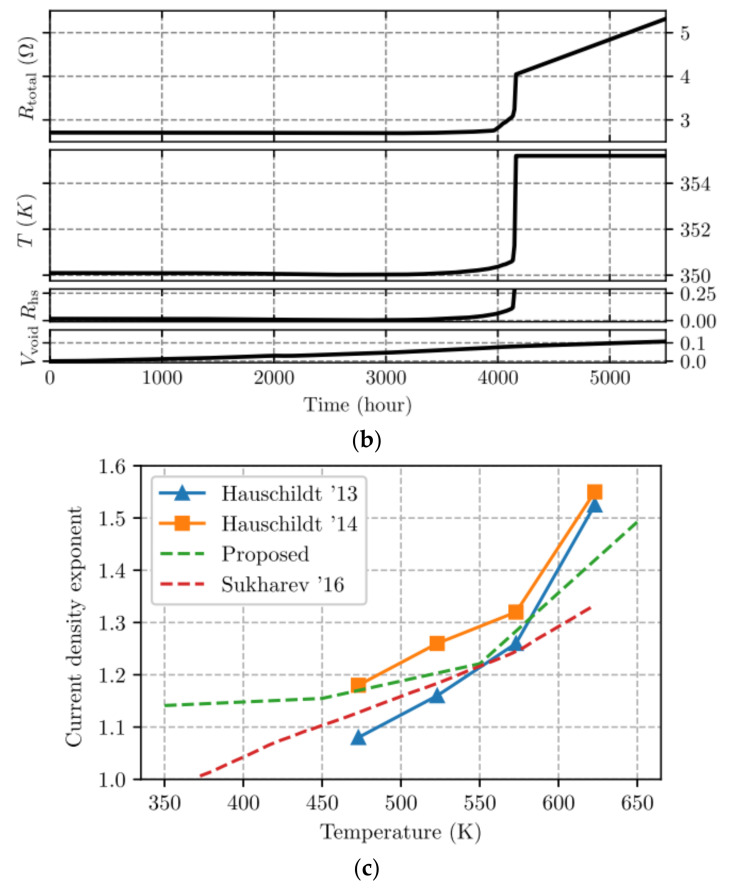
(**a**) 3D illustration of up-stream interconnect structure and simulated physical systems; (**b**) simulation of Joule heating effect. R_total_: total wire resistance. T: temperature at the hotspot. R_hs_, in ohms: copper resistance at the hotspot. Void, in cubic micrometer: simulated void size; (**c**) extracted current density exponent compared to experiment data and previous postvoiding EM analysis work [85,86].

EM postvoiding analysis attracts researchers’ attention because it is hard to handle the interactions between current density, hydrostatic stress, and temperature when the target is to investigate detailed void growth. Hengyang Zhao et al. proposed a multi-physics finite-element-method-based (FEM-based) analysis method for void growth simulation in confined copper interconnect [85,86]. The method considers time-varying interactions between hydrostatic stress in the confined interconnects structure illustrated in Figure 11a, the current density and Joule heating induced temperature rise. The interactions are realized by solving a set of coupled partial differential equations, including the Korhonen’s equation, the phase field equation, the Laplace equation, and the heat diffusion equation. The FEM-based EM solver is capable to predict unique transient resistance change for copper interconnects. Figure 11b shows the time-dependent total resistance, hotspot temperature, resistance at the hotspot, and void size. It verifies the statement in Section 2.2 that the resistance jump between nucleation phase and growth phase is due to Joule heating. Meanwhile, the lifetime distribution from this EM solver can provide a higher fitting accuracy on the current density exponent parameter (*n*) described in Black’s equation in the previous study, as shown in Figure 11c. 

**Figure 12:**
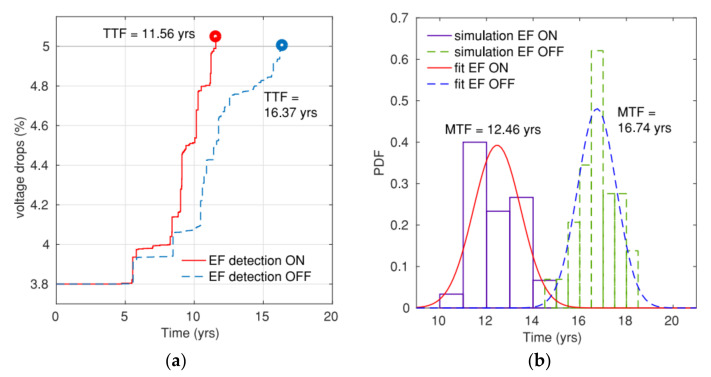
Impact of early failures on (**a**) voltage drop of a sample grid and (**b**) estimated mesh MTF for ibmpg2 [44].

**Figure 13:**
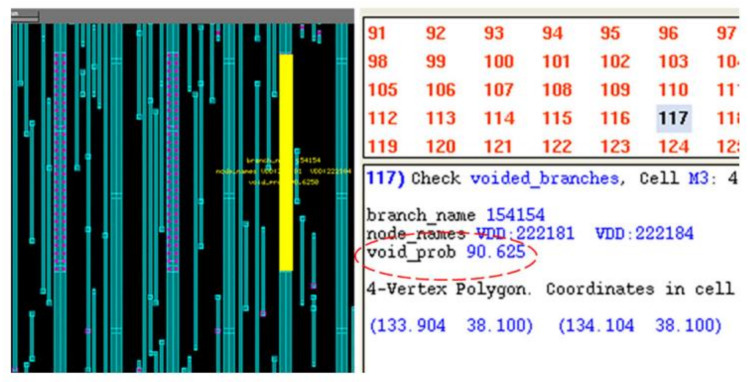
Highlighted wire with high voiding probability on the layout of a metal layer [91].

Sandeep Chatterjee et al. also proposed a methodology to check P/G EM by using physics-based model [41,44]. They firstly listed the detailed steps to extend the physical models for EM in branches to compute the hydrostatic stress evolution in multi-branch interconnect trees. The boundary condition for branches under different scenarios are provided as well. Then filtering and predictor-based schemes are designed to speed up the overall EM assessment. It enables the statistical computation on IBM benchmarks finish in 2.3 hrs. Therefore, the proposed method has potential to be applied on large-scale circuits. The simulation results verified the inaccuracy of Black’s model and explained the importance of *early failures* for EM assessment. As shown in Figure 12, exclusion of early failures leads to optimistic evaluation on voltage drop increase and on MTF of P/Gs [44]. In [87], a finite difference method-based EM analysis methodology is applied to 3-D IC test structure. Its comparison with finite element analysis and experiment measurement demonstrates that EM in 3D IC structures can be suitably evaluated with finite difference simulation. In [88], the authors presented a systematic approach to resize the grid metal lines to achieve a design target lifetime at the minimal extra cost of metal area. With the help of this approach, on a grid with 1.2 million nodes, the authors can increase its MTF from 10.5 y to 12.2 y under a cost of 0.02% extra metal area by scaling only 14 interconnect trees. Current density variation is an important factor for EM evaluation. To ensure the comprehensiveness of their EM modeling, Adam Issa et al. has investigated EM checking by using stochastic effective current model [89]. It is observed that current variations bring us worse EM reliability. Based on his EM modeling experience, Farid N. Najm derived the equivalent circuits for EM under different model phases [90]. It is shown that the dynamic behavior of stress and flux in metal line is identical to dynamic behavior of voltage and current in RC circuit, thus EM assessment can be executed by simulating its equivalent RC circuit. It has potential to drastically improve EM assessment capability on large circuits. In [91], an industry-level physics-based tool for EM assessment in commercial-grade PDNs was introduced. As shown in Figure 13, after analysis the tool can highlight voided metal line segments with a voiding probability. The tool’s accuracy has been validated with the experimental data [92]. There is good fit between lifetime statistics derived from measurement (*MTTF_EXP_* = 62,305 s, Δ_EXP_ = 14,012 s) and simulation (*MTTF_SIM_* = 60,344 s, Δ_SIM_ = 12,613 s). 

Meanwhile, Houman Zahedmanesh et al. investigated the EM limits of Cu nano-interconnects by using a novel hybrid physics-based model [93]. The modeling framework incorporates variations of materials, dimensions, interfaces, and operation conditions. It considers void dynamics and resistance shift by using a local cellular automation module with a resistive network. The simulation result only shows that the nucleation phase gets more significant under a narrower linewidth, it but also predicts complex R-shift signatures which match well with experimental data. Sarath Mohanachandran Nair et al. proposed a variation-aware physics-based EM modelling which is experimentally calibrated [94]. The model can be used to explore the impact of material and dimension on design space and to study failure time variation at various operating conditions. Then the model was extended to handle both *early* and *conventional failures* [95]. In [96,97], the system-level simulation on EM under 3 nm technology node was performed. It is shown that Ru rails reduced IR-drop penalty by a factor of ~0.6 than the Cu rails. Although EM voids appear in multiple PDN segments, the EM induced IR-drop always stay below 3.3% without any failed operation of standard cells.

**Figure 14:**
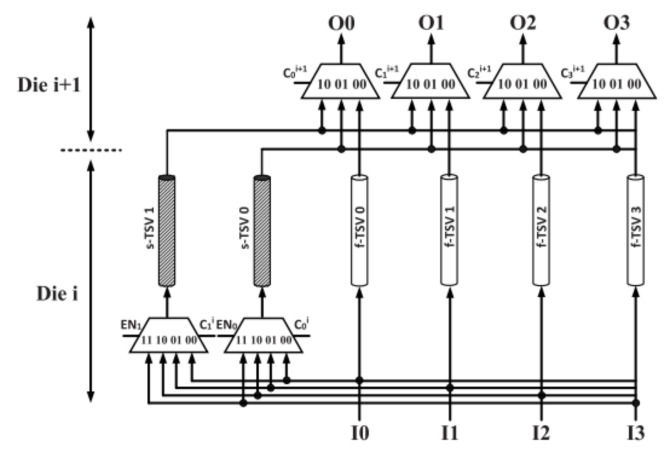
The reconfigurable routing network for a (4:2) TSV group consisting of four f-TSVs and two s-TSVs [100].

Except for the studies on EM reliability of full power grids, there are other novel works on EM by using physics-based model. In [98,99,100], the authors explored an approach to enhance the TSV grid reliability. The main idea is to allow the nonfaulty TSVs to be temporarily deactivated so that it can take advantage of EM recovery property. To achieve this goal, a reconfigurable routing network for a (4:2) TSV group was adopted, as depicted in Figure 14. Depending on a recovery schedule, all TSVs cab operate under active mode, recovery mode. The recovery-aware proactive repair approach helps improve EM lifetime of the entire TSV grid by up to 12 times relative to conventional reactive method without an extra area cost. 

### 3.2. Fast EM Assessment and Optimization

**Figure 15:**
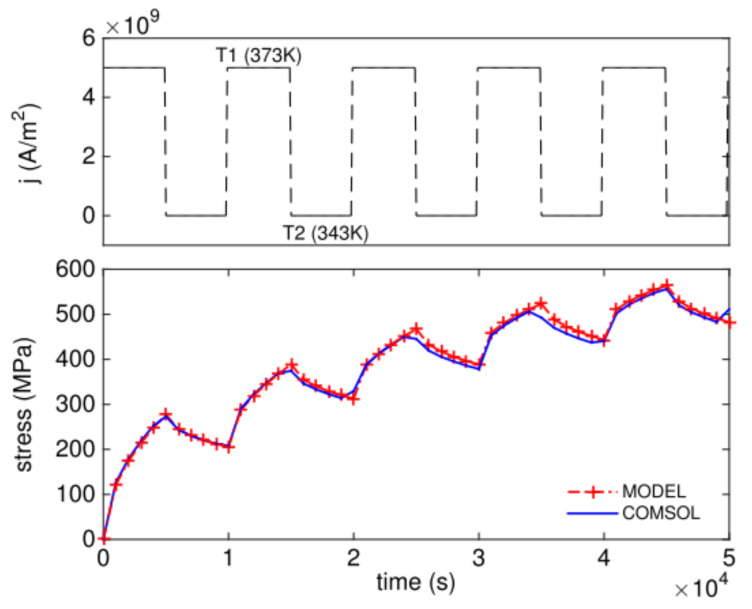
Stress evolution caused by periodic unipolar pulse current densities at cathode end of the metal line under varying temperature [103].

Although FDM and FEM methods can be applied for transient EM analysis with good accuracy, they are not always preferred when the studied interconnect structures are too large or the computation resource is limited. To overcome this problem, a number of analytical and semi-analytical solutions and speed up techniques have been proposed. At the very beginning, Valeriy Sukharev and Xin Huang et al. derived the analytical equation for transient hydrostatic stress in a confined metal wire [101,102,103]. The analytical equation captures the impact of time-dependent current density and the stress recovery effect, as shown in Figure 15. The calculation results not only show that in the case of high frequency currents with periods much smaller than the characteristic time of stress evolution, the pulse duty factor decides stress buildup, it but also demonstrates that temperature oscillation can cause notable resistance increase in a short metal line with preexisted voids. On the other hand, the stress recovery effect is applicable to improve on-chip interconnect lifetime by properly managing driving powers at run time. 

Later, Hai-Bao Chen et al. developed a first principle based analytical solution for hydrostatic stress evolution in 3 specific interconnect trees [104,105]. It solves stress evolution in multi-branch tree by de-coupling individual branches with suitable boundary conditions which account for interactions between adjacent branches. The solution is based on Laplace transformation technique. Since time-varying temperature and current density and branch length have non-negligible impact on stress evolution, they incorporated these factors into their analytical solution under the same method [106,107]. However, since these analytical solutions only work for specific tree structures, Xiaoyi Wang et al. applied eigenfunction technique for stress evolution in multi-branch trees, and they have extended it to EM analysis in full-chip P/Gs [108,109,110]. This method handles different current densities and nonuniform thermal distribution. Since this method does not require discretization, except for its excellent scalability for large-scaled interconnect trees, it brings 10–100 times of computation speed improvement over FDM method. In order to improve EM analysis speed, Liang Chen et al. proposed accelerated separation of variables (ASOV) method which offers improvements over the existing plain SOV-based method [111]. It exhibits 3–5 times of speedup on a number of multi-branch interconnects benchmarks. Furthermore, the SOV-based approach was adopted to obtain a semi-analytical stress transient analysis method which considers the impact of temperature gradient in the studied trees [112]. This method is about an order of magnitude faster than COMSOL with 10× less memory footprint and negligible error loss. It shows that the impact of Joule heating on EM process is significant. Mohammadamir Kavousi et al. studied EM immortality check with considering Joule heating for multi-branch trees [113]. They improved EM stress analysis speed with the benefit of Krylov subspace-based reduction technique which reduces the size of system matrices [114]. Their analysis on interconnect with up to 1000 branches for both void nucleation and growth phases has been accelerated by 28 times. Last, but not least, Mohammad Abdullah Al Shohel et al. found a linear-time approach for immortality check on general tree/mesh interconnects and developed an analytical model of transient stress based on boundary reflections [115,116]. With respect to speedup techniques different from analytical solution, Sandeep Chatterjee et al. presented a fast and scalable methodology for P/G EM verification [117]. The PDE system was converted to a succession of homogeneous linear time invariant (LTI) system. Then the LTI system was solved with an optimized backward differentiation formulas (BDF) solver. Under further help from preconditioned conjugate gradient and parallel programming, this method gives around 23 times of speedup. In [118], a Krylov subspace-based method was proposed for fast stress evolution under finite difference method. After discretization, the original system matrices are reduced so that they can be simulated more efficiently in time domain. This optimized method brings 1–2 orders of magnitude speedup over ordinary finite difference time domain methods. 

**Figure 16:**
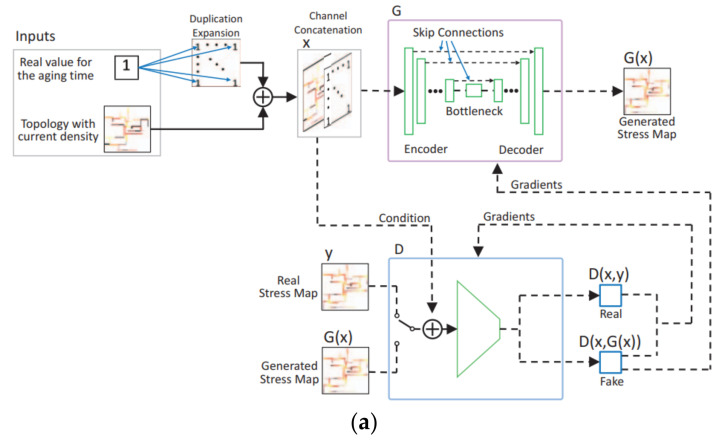

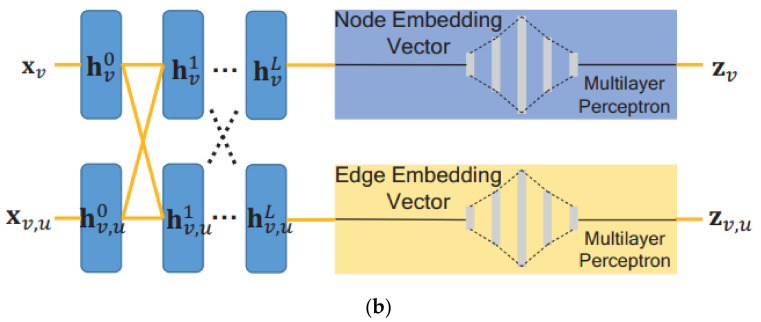
(**a**) EM-GAN framework for stress estimation and (**b**) framework of EMGraph with multilayer perceptron network [119,120].

Recently, machine learning techniques have been applied to speed up studies on P/G performance and reliability. In [119], a generative adversarial networks-based (GAN-based) tool (called EM-GAN) was built to do fast analysis on transient stress in multi-branch trees. This work was inspired by the image synthesis feature of GAN. As shown in Figure 16a, the GAN’s inputs include P/G topology, current density distribution at a given aging time. Its output is EM stress distribution. This tool can achieve high prediction accuracy with an average error of 6.6%, and it exhibits 8.3 times speedup over analytic EM solver. In order to achieve even higher accuracy, another graph convolution network-based (GCN-based) tool (called EMGraph) was developed for transient EM stress estimation. Its basic framework is shown in Figure 16b. It shows less than 1.5% average error compared with training data and orders of magnitude faster than COMSOL. Moreover, EMGraph surpasses EM-GAN with 4 times higher prediction accuracy and 14 times faster speed. 

**Figure 17:**
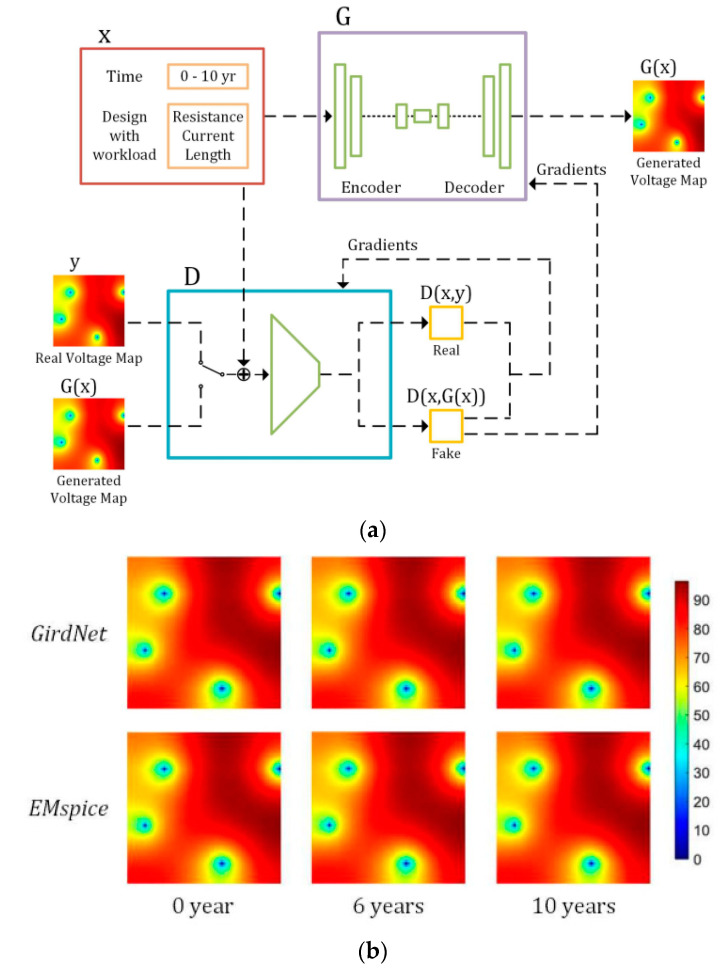
(**a**) CGAN architecture for EM-induced voltage prediction and (**b**) comparison of inference results from GridNet to EMSpice [121].

In [121], a conditional generative adversarial networks-based (CGAN-based) framework (called *GridNet*) was developed, as shown in Figure 17, to accelerate the incremental full-chip EM-induced IR drop analysis and the optimization for IR drop violation fixing. GridNet provides accurate prediction on IR drop as compared with the ground truth obtained from EMSpice. Since GridNet also provides sensitivity information of node voltage with respect to branch resistance, it expediates localized IR drop violation fixing for P/G design. 

Successful P/G design targets at a good enough EM lifetime with a reasonable cost on area. P/G design optimization is an important process to get a balance between EM reliability and area cost. Han Zhou et al. proposed P/G optimization techniques based on fast EM nucleation phase immortality check method for multi-branch interconnect trees [122,123]. They, firstly, verified that the issue can be formulated as a sequence of linear programming problem. Then they proposed an aging-aware optimization method which improves mortal wires’ lifetime by adding reservoir branches and allows some interconnects to age/breakdown then just optimizes EM reliability of remaining branches. This strategy ensures the optimization operate effectively. Numerical results demonstrated that the new method can effectively reduce P/G area while ensuring immortality or target lifetime of all the wires. Later, the EM incubation phase immortality check method is introduced into the optimization framework as well [124,125]. The updated method can fix IR drop violation due to EM in minutes for P/Gs from ARM core designs. 

### 3.3. EM Acceleration

**Figure 18:**
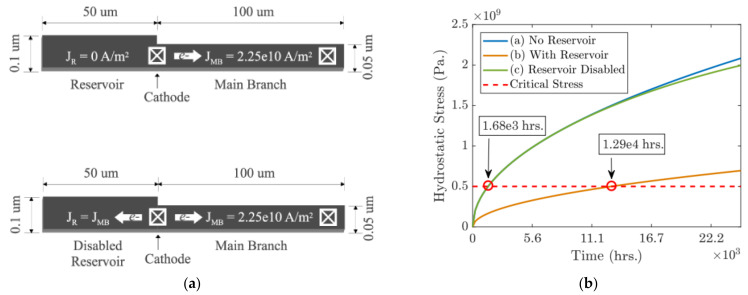
(**a**) An active interconnect branch with a reservoir branch enabled and disabled and (**b**) Hydrostatic stress at cathode under various scenarios [128].

In order to effectively validate the reliability of dual damascene interconnect trees under specific process and structure, it is necessary to detect EM failures in a relatively short testing time. Since the traditional acceleration techniques mainly focus on high temperature and current density, they lead to higher probability of failure due to not only EM but also the other aging mechanisms such as BTI and HCI, which leads to less distinguishability between the mechanisms. In [126,127,128], several techniques have been proposed to accelerate and decelerate EM failure with the help of reservoir branches and sink branches. Figure 18 shows an active branch with a reservoir branch enabled/disabled and the time-dependent hydrostatic stress at cathode. Obviously, the reservoir branch highly extends void nucleation time at the cathode which indicates much better EM reliability. The impact of reservoir could be disabled by applying a current to it in a reversed direction. And the current density decides how much the main branch’s lifetime is suppressed. 

**Figure 19:**
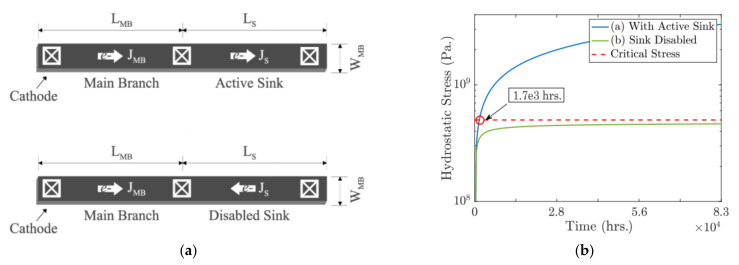
(**a**) An active interconnect branch with a sink branch enabled and disabled and (**b**) hydrostatic stress at cathode with the sink branch enabled and disabled [128].

In [127,128], a sink branch was proposed to accelerate EM lifetime of the main branch. As shown in Figure 19, the void nucleation can be accelerated by an active sink branch because the atomic flux gets improved when the currents over main branch and sink are in the same direction [128]. Then the authors proposed a hybrid structure with both sink and reservoir applied to accelerate EM failure. The proposed structures can achieve desired very short TTF under acceleration mode while the main branch itself has 10+ y lifetime under normal usage mode. These novel structures together with temperature control can further accelerate EM testing about 10^5^ time under the 150 °C temperature limit. 

## 4. Modeling of EM Impact on Interconnects in Cache Memory

**Figure 20:**
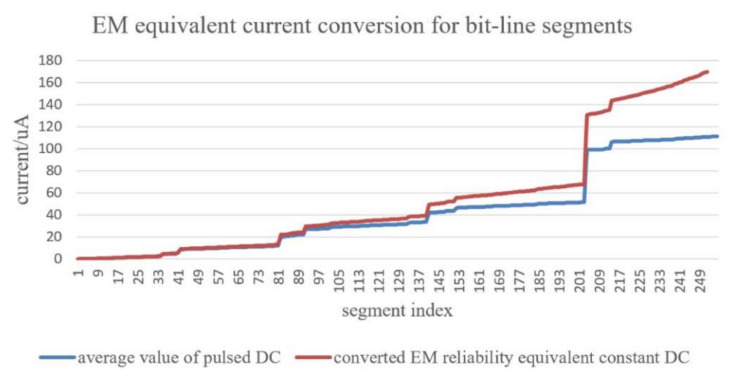
Typical current distribution comparison between time-average value and converted value for all segments within an SRAM bitline [131].

**Figure 21:**
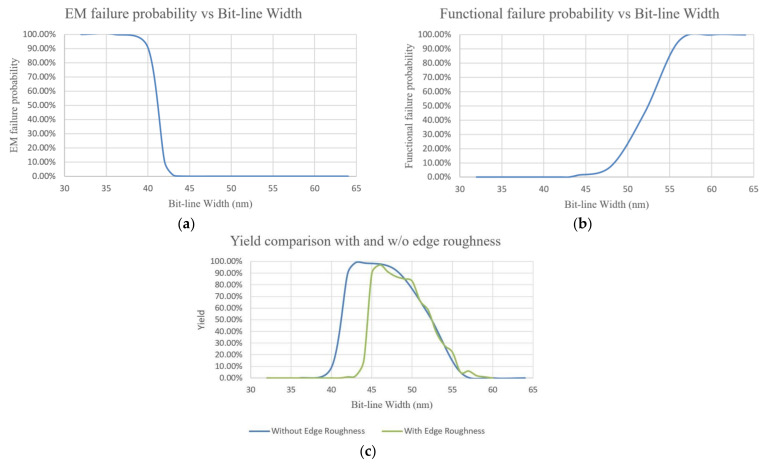
(**a**) EM probability of failure, (**b**) functional probability of failure, and (**c**) yield comparison for 256 rows × 128 columns 22-nm SRAM array w/and w/o considering edge roughness [131].

EM not only affects P/G but also worsen the reliability of bitlines which is frequently stressed by currents during read/write operations of cache memory. During read/write operations, the unbalanced currents flowing over interconnects cause voids which ultimately lead to operation failure by introducing large delay. In [129,130,131], the authors designed a methodology for SRAM EM reliability assessment with considering process variations. The equivalent current distributions over bitlines are calculated with an AFD-based current conversion scheme. As shown in Figure 20, the equivalent current is much different from average value of pulsed DC which indicates different conclusion on EM reliability. The current distribution is combined with process variations including threshold voltage variation, gate length variation, and bitline edge roughness, to evaluate bitlines’ reliability by using statistical modelling methodology. The authors adjusted bitline width to find an optimal value to minimize the total probability of failure for an SRAM array and to maximize its yield. The experiment results indicate that a 22 nm technology-based 256 rows × 128 columns SRAM array suffer from serious EM issue if the bitline width is chosen as ½ metal pitch, as shown in Figure 21. And a tradeoff between functional failure and EM failure can be reached for a 46 nm width bitline when the edge roughness is incorporated.

The current distributions over bitlines are decided by activity of cells attached to them. The impact of cell activity on EM reliability of an SRAM cell array was studied in [132]. However, since none of the previous prior works have taken SRAM workload in realistic usage scenarios to evaluate interconnects’ reliability, a simulator called CacheEM was proposed for reliability analysis on cache memory aging due EM by considering the realistic application scenarios of cache in an ARM microprocessor [49]. CacheEM includes five parts: microprocessor emulation, memory cell array activity extraction, computation of current in long interconnects, evaluation on time-dependent hydrostatic stress and the resistance shift of interconnects, and characterization of the interconnect EM lifetime distribution of a cache memory.

**Figure 22:**
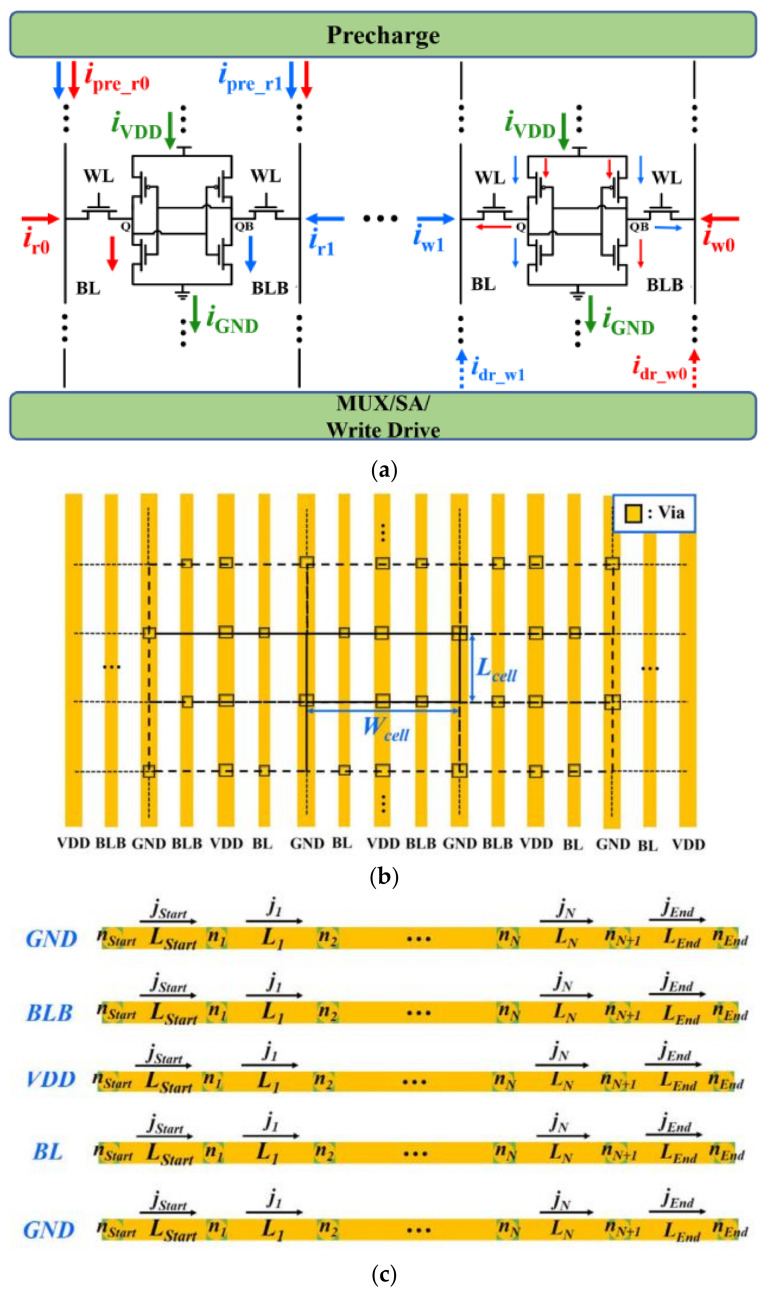
(**a**) Simplified schematic of SRAM cell array with currents relevant with various operation marked. The left and right parts show the currents relevant with read 0 and 1 and write 0 and 1, respectively. The currents for read 0 and write 0 in red, while the currents for read 1 and write 1 are in blue, (**b**) interconnect array in SRAM cell array, with periodic cells shown functional probability of failure, and (**c**) representative interconnects corresponding to a column of cells [49].

**Figure 23:**
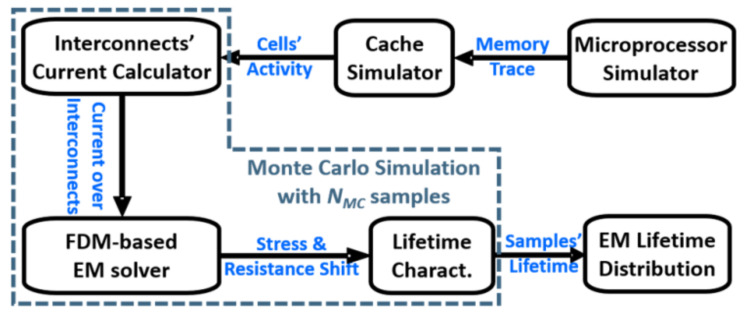
Simulation flow of CacheEM [49].

Figure 22a shows a simplified schematic of an SRAM cell array which is handled in CacheEM with currents corresponding to specific operations (read 0 and1 and write 0 and1) marked. Figure 22b,c show interconnect-array in SRAM cell array and the representative interconnects corresponding to a column of cells [49]. *L_Start_* and *L_End_* are the segments from the array to the pre-charge and write drivers, respectively. In order to perform time-dependent EM assessment on these interconnects, the current density distributions over them need to be determined in the first step. To obtain the current density distributions, it is required to know the number of load and store to each cell, which means the cells’ activity in cache memory needs to be extracted. On the other hand, the activity of cells in the cache memory highly depends on the application of the microprocessor where the cache is configured. It needs a simulation flow for CacheEM from a top–down perspective as described in Figure 23. In the first step, several testbenches on the microprocessor which is configured with specific cache memory settings are executed. The trace of the target cache memory is recorded. In the second step, the recorded memory trace is fed into a cache simulator to extract the cells’ activity. Then, the variations in effective atomic diffusivity and threshold stress are incorporated into CacheEM with a Monte Carlo simulation to obtain the cache EM lifetime distribution. For each sample, the current flowing in each branch is derived individually with considering the cells’ activity and the currents during each operation (read 0/1 and write 0/1). 

**Figure 24:**
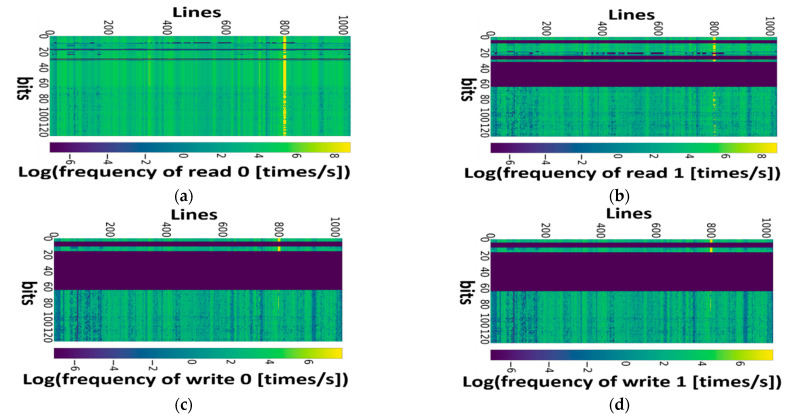
(**a**) Read 0, (**b**) read 1, (**c**) write 0, and (**d**) write 1 distributions of L1 I-Cache cells in ARMv8 core which has run sjeng, specrand, and *patricia* benchmarks [49].

**Figure 25:**
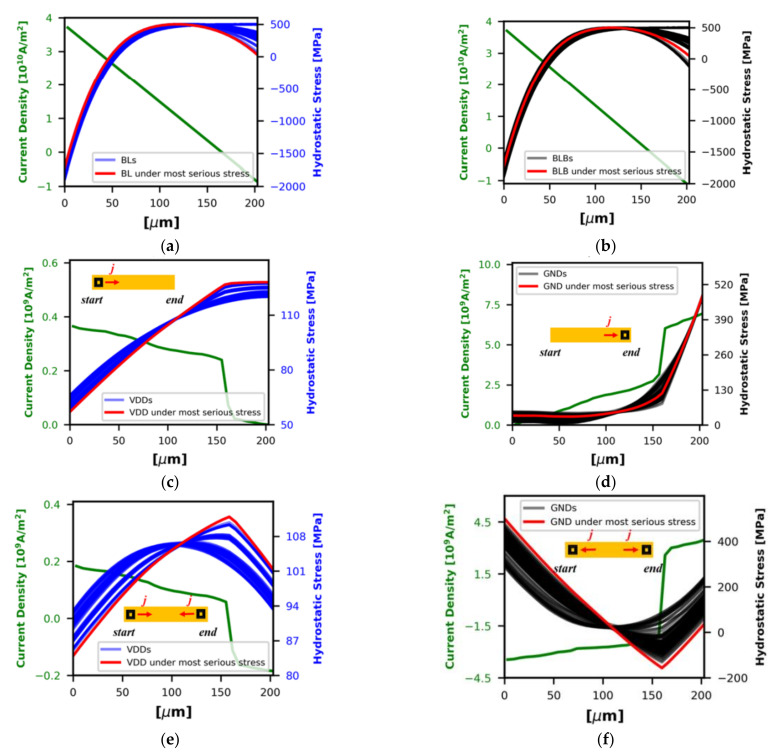
The hydrostatic stress distribution before a void appears in (**a**) BLs, (**b**) BLBs, (**c**) VDDs in a one port implementation, (**d**) GNDs in a one port implementation, (**e**) VDDs in a two-port implementation, and (**f**) GNDs in a two-port implementation [49].

Figure 24 shows the representative operation distributions on the L1 I-Cache cells in an ARMv8 core while it is running the *sjeng*, *specrand*, and *patricia* benchmarks [49]. The cells’ activity and currents during read/write operations under user-defined parameters, including gate length, bit-line unit capacitance, row number of the cells, supply voltage, and temperature are combined together to calculate the effective current density distributions over each BL, BLB, VDD, and GND interconnects. Then they are provided to FDM-based EM solver to evaluate time-dependent stress evolution. The EM lifetime distribution is measured under predefined threshold value. Figure 25 shows the hydrostatic stress distribution of VDDs, GNDs, BLs, and BLBs in randomly selected caches [49]. The current density in the interconnect which suffers the most serious EM stress is plotted on the basis of the left *y*-axis in each subplot. In BL and BLB, the voids are most likely formed in the middle of the lines. The exact void position depends on the ratio between read and write operations. The voids most likely appear at the ends of VDDs and GNDs. However, the voids most likely appear near the via for GNDs, but far from the vias for the VDDs due to the difference of current direction. Stress is highest when current flows into the vias, and lowest when current flows away from the vias. Under a two-port implementation and bi-directional flow, the stress is highest around the middle of the VDDs and at the ends of the GNDs. After the maximum stress reaches *σ_th_*, the voids are generated, and then after the void size becomes larger than the threshold size, the interconnect resistance increases. An interconnect’s lifetime is obtained when the resistance shift reaches a pre-defined threshold value. 

**Figure 26:**
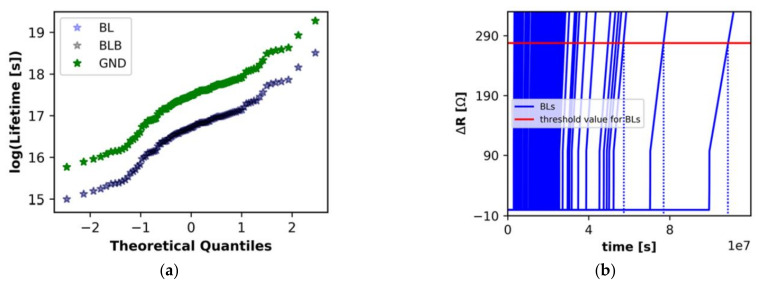
(**a**) Lifetime distribution of the BL, BLB, and GND interconnects which suffer from the most serious stress in samples of an I-Cache, and (**b**) the time-dependent resistance shift of the corresponding BLs [49].

Figure 26a shows the lifetime distribution of BL, BLB, and GND interconnects which suffer from the most stress in 100 samples of an I-Cache. With the help of the two-ports connection, GND has a much better EM reliability than BL and BLB. BL is the most vulnerable interconnect in the I-Cache. Figure 26b shows the time-dependent resistance shift of the corresponding BLs in 100 cache samples [49]. BL is not always more vulnerable than BLB. Their relative vulnerability might change with the cache configuration. CacheEM was applied to investigate the impact of configuration parameters on performance and EM degradation of an I-Cache. The configuration parameters include cache line size, replacement policy, set associativity, latency, and cache size. Cache line size has options of 8B, 16B, and 32B. The I-Cache hit rate is 94.43%, 93.65%, and 93.75%, when the line size is 8B, 16B, and 32B, respectively. 32B brings better lifetime than the other two options because the interconnect length is much shorter while the current density is smaller. Replacement policy, associativity, and latency do not have obvious impact on I-Cache performance and reliability. Cache size has a non-negligible impact on the hit rate and the lifetime distribution of the studied I-Cache. The hit rate is 89.16% under 8 KB, 93.65% under 16 KB, 97.39% under 32 KB, and 99.19% 64 KB, respectively. 8 KB cache has better lifetime than the other three options because the interconnect length is shorter than the other cases and the current density is smaller. For the remaining three cases, EM reliability improves with the increase of the cache size.

## 5. Conclusions

Recent progress in physics-based modeling of EM aging of on-chip interconnects has been reviewed in this paper. Due to the over-conservative EM assessment on multi-branch interconnect tree by conventional Black’s equation, physics-based modeling methodologies which consider the atom flow between adjacent branches are required to provide more accurate EM assessment. These modeling methodologies can be achieved with analytical equations or numerical computations. Voltage-based immortality check can simplify EM assessment by eliminating the immortal trees which do not need time-dependent evaluation on stress and resistance. The speedup techniques, such as separation of variable method and Krylov subspace-based reduction technique, are applicable for fast assessment on large-scaled circuits. Machine learning algorithms like GAN also help improve assessment efficiency. With respect to interconnects in cache memory, their EM reliability depends on physical dimensions, cache configurations, and workloads under specific benchmarks. 

Since the integration density in SoC is always getting higher to achieve better system performance, the number of interconnect trees to be studied becomes tremendous. It is a challenging task to evaluate the full EM reliability of on-chip interconnects with good accuracy as well as fast speed. Further application of machine learning techniques deserves more attention to accelerate the evaluation speed and to improve the models’ prediction capability. On the other hand, since Cu is predicted to be replaced by novel materials such as Co and Ru to ensure interconnects’ reliability at sub-10 nm technology nodes, more efforts need to be made to novel modeling methodologies which suitably estimate the aging of new material-based interconnects and networks.
